# The need for particular scrutiny of claims made by researchers associated with ultra-processed food manufacturers

**DOI:** 10.1017/S0007114523000429

**Published:** 2023-10-28

**Authors:** Mark Lawrence

**Affiliations:** Institute for Physical Activity and Nutrition, Deakin University, Burwood, VIC 3125, Australia

Dear Editor

Thank you again for your invitation for me to provide a Commentary on ultra-processed foods (UPF)^(1)^. In this Commentary, I referred to challenges the UPF concept presents to researchers with declared associations with UPF manufacturers. The interplay between nutrition research and commercial interests is a widely recognised phenomenon in the commercial determinants of health literature. For example, it has been reported that findings from systematic reviews of sugar-sweetened beverage consumption and weight gain in which the researchers’ declared interests with UPF manufacturers were less likely to be counter to the sponsor’s interests than reviews on the same topic where no interests were declared^(2)^. Of course, not all researchers declaring associations with UPF manufacturers make claims to adversely influence policy processes. Nevertheless, UPF-related research has become highly politicised and the integrity of the claims presented by researchers associated with UPF manufacturers demands close scrutiny.

It was therefore surprising that in a letter in reply to my Commentary, published in this issue of the journal, Messina *et al*. accuse me of using ‘classic ad hominem reasoning’ in describing this phenomenon^(3)^. This is an extraordinary accusation and it is incorrect as there was no personal attack anywhere in my Commentary. It is also baseless for reasons which include:

1. Messina *et al.* misrepresent the Commentary’s purpose

The Commentary’s purpose was to describe the politicised nature of UPF-related research and call for close scrutiny of claims being made by researchers with declared interests with UPF manufacturers. Messina *et al.* appear to be aggrieved that in their view the purpose of the Commentary should instead have been only to focus specifically on technical aspects of their argument that the UPF concept should not apply to soya-based meat and dairy alternatives. As it happens, the Commentary did address this argument and it did so in the context of the Commentary’s purpose. It noted the argument had not compared the broader public health, environmental and social implications of UPF plant-source protein foods with existing non-UPF nutritious plant-source protein foods such as legumes and nuts. This is a relevant statement because the broader analytical scope provides a more complete assessment of the health, sustainability and social implications of these UPF products. Unfortunately, Messina *et al*. misrepresented the statement when responding, ‘the products in question are designed to replace meat and dairy not legumes and nuts’. The Commentary did NOT refer to soya-based meat and dairy alternatives as replacements for legumes and nuts. Instead, it referred to legumes and nuts as non-UPF alternatives to UPF plant-source protein foods in replacing meat and dairy foods.

Messina *et al.* dismiss the Commentary’s narrative by claiming that soya-based meat and dairy alternatives and not legumes and nuts are the logical replacements for meat and dairy foods. This comparison is questionable from a practical and nutrition science perspective. Minimally processed legumes and nuts have a long history of being partial, if not complete, replacements for meat in many traditional dietary patterns. Moreover, a recent Nordic study reports that meat can be at least partially replaced by legumes with no adverse impact on nutrient intake and nutrient adequacy^(4)^.

In addition, Messina *et al*. ignore that it is the overall ratio of plant-source to animal-source foods in the diet that is the core healthy and sustainable diet recommendation and not the replacement of specific animal-source foods with specific plant-source foods.

The Commentary was agnostic towards the potential benefits and risks of alternative plant-source protein foods as replacements for meat and dairy products. This is a complex and nuanced area. But what is clear is that there are non-UPF soyamilk products and non-UPF plant-sourced burger patties available as dairy and meat replacements. Their availability makes the need for UPF versions of such products questionable. It is strange that in claiming they are arguing for healthy and sustainable diets Messina *et al.* appear to be more concerned with challenging the UPF concept than promoting already available non-UPF foods.

1. It is factually accurate to note that some of the authors have declared associations with UPF manufacturers.

My noting of Messina *et al.*’s declared associations with UPF manufactures is accurate and not even novel. For example, previously Nestle has commented on this arrangement when reviewing the paper cited in the Commentary, and she also noted that the authors themselves make it clear that they have a very long list of declared associations with UPF manufacturers^(5)^.

These reasons may be inconvenient to the authors’ argument, but that does not justify them making a baseless claim of ad hominem reasoning against me. A particular concern is that the authors’ accusation risks diverting attention away from the Commentary’s narrative that ‘*the integrity of the claims presented by researchers with UPF associations demands close scrutiny*’. It would be interesting to examine if making baseless accusations of ad hominem reasoning have the effect of ‘chilling’ such scrutiny. Scrutiny of the integrity of all nutrition claims is a strength of nutrition science practice. This experience serves to illustrate why particular scrutiny of the integrity of nutrition claims of researchers with associations with UPF manufacturers is timely and important.
